# Exploiting Layered Multi-Path Routing Protocols to Avoid Void Hole Regions for Reliable Data Delivery and Efficient Energy Management for IoT-Enabled Underwater WSNs

**DOI:** 10.3390/s19030510

**Published:** 2019-01-26

**Authors:** Suhail Ashfaq Butt, Kamalrulnizam Abu Bakar, Nadeem Javaid, Niayesh Gharaei, Farruh Ishmanov, Muhammad Khalil Afzal, Muhammad Khalid Mehmood, Muhammad Akram Mujahid

**Affiliations:** 1 School of Computing, Universiti Teknologi Malaysia (UTM), Skudai 81310, Johor, Malaysia; suhail.ashfaq@ue.edu.pk (S.A.B.); knizam@utm.my (K.A.B.); niya.gharaei@gmail.com (N.G.); momikh@gmail.com (M.K.M.); akram.mujahid@ue.edu.pk (M.A.M.); 2 Division of Science and Technology, University of Education, Lahore 54000, Pakistan; 3 Department of Computer Science, COMSATS University Islamabad, Islamabad 44000, Pakistan; 4 Department of Electronics and Communication Engineering, Kwangwoon University, Seoul 01897, Korea; 5 Wah Campus, COMSATS University Islamabad, Wah Cantonment 47040, Pakistan; khalilafzal@ciitwah.edu.pk; 6 Cholistan University of Veterinary and Animal Sciences, Bahawalpur 63100, Pakistan

**Keywords:** retransmission, energy efficient, void hole, energy hole, multi-path layered approach, cross nodes, reliable data delivery

## Abstract

The key concerns to enhance the lifetime of IoT-enabled Underwater Wireless Sensor Networks (IoT-UWSNs) are energy-efficiency and reliable data delivery under constrained resource. Traditional transmission approaches increase the communication overhead, which results in congestion and affect the reliable data delivery. Currently, many routing protocols have been proposed for UWSNs to ensure reliable data delivery and to conserve the node’s battery with minimum communication overhead (by avoiding void holes in the network). In this paper, adaptive energy-efficient routing protocols are proposed to tackle the aforementioned problems using the Shortest Path First (SPF) with least number of active nodes strategy. These novel protocols have been developed by integrating the prominent features of Forward Layered Multi-path Power Control One (FLMPC-One) routing protocol, which uses 2-hop neighbor information, Forward Layered Multi-path Power Control Two (FLMPC-Two) routing protocol, which uses 3-hop neighbor information and ’Dijkstra’ algorithm (for shortest path selection). Different Packet Sizes (PSs) with different Data Rates (DRs) are also taken into consideration to check the dynamicity of the proposed protocols. The achieved outcomes clearly validate the proposed protocols, namely: Shortest Path First using 3-hop neighbors information (SPF-Three) and Breadth First Search with Shortest Path First using 3-hop neighbors information (BFS-SPF-Three). Simulation results show the effectiveness of the proposed protocols in terms of minimum Energy Consumption (EC) and Required Packet Error Rate (RPER) with a minimum number of active nodes at the cost of affordable delay.

## 1. Introduction

In recent years, UWSN has acquired enough attention of researchers to encapsulate a wide range of applications, i.e., marine life monitoring, oceanic data collection, disaster prevention, monitoring, etc. [1]. UWSNs mainly consist of different types of nodes, channels, noises, etc. These nodes collect the data packets from the source node by facing different noises and forward them to the nearest sink placed at the surface using different channels. Radio waves used in the terrestrial environment cannot be used in UWSNs because radio waves get quick attenuation, resulting in short propagation distance. Therefore, acoustic signals emerge as a suitable choice for communication in water because of low absorption rate.

Acoustic signals have low absorption rate and face a high end-to-end delay because of low propagation speed in UWSNs. In addition, different noises, doppler effect and path losses introduces high EC and Bit Error Rate (BER). This BER decreases the reliability of data packets during transmission which results in high EC [2].

According to the authors in [3], acoustic signals are powerful near the signal’s origin and get attenuated from noise. Additionally, noise intensity is minimum in deep water and maximum in shallow water. Therefore, minimum path distances eliminate the problem of packet redundancy and high energy dissipation which result in fewer collisions and packet retransmissions. Sensor node’s battery life is an important factor for routing in UWSN. An energy-efficient routing protocol is required to minimize the EC, maximize the data collection, and enhance the reliability of data with affordable delay. The main objectives of protocol designing include:

- Energy-efficient network
- Maximum data collection
- Reliable data delivery
- Affordable delay
- Required packet received ratio
- Efficient route selection with least number of active nodes

Many researchers proposed different protocols to enhance the capabilities of UWSNs. For instance, efficient sink mobility is presented in [4], reliable data transmission is achieved in [5] and direct forwarding mechanism is proposed to reduce void holes and EC [6,7]. Linear forwarding is adopted with single path and multiple paths to transmit data packets from source to destination for minimal EC [8,9]. In the same way, geographical routing is globally accepted due to its scalability and simple implementation methodology. Somehow, if these packets survive from energy holes, different noises existing in their path make the packets erroneous. Therefore, data is sent through multiple paths using binary tree generation which decrease the chances of data corruption with possible energy overhead.

In this work, two forwarded multi-path power control protocols are proposed: SPF-Three and BFS-SPF-Three. These protocols forward the data packets with the aim of eliminating retransmissions. It also minimizes the EC and RPER with an affordable delay to avoid the void holes. End-to-end delay is increased due to retransmissions. These retransmissions effect the performance of underwater network in terms of minimum Packet Received Ratio (PRR). To encapsulate the EC problem, network is divided into different layers by adopting the shortest path towards the destination. Data packets are uni-casted from relay nodes and multi-casted from cross nodes. This idea follows a greedy approach for transmitting the data towards the destination. Proposed protocols confirm 3-hop neighbors for data transmission and after confirmation, they choose the next hop neighbor as a current forwarder. This selection of next hop helps in avoiding the void hole regions to prolong the network lifetime with reliable data delivery. Abbreviations and acronyms with their notations are mentioned in Table 1 and Table 2, respectively. The main contributions of proposed protocols are summarized below:

- Two routing protocols are proposed: SPF-Three and BFS-SPF-Three
- Avoid void hole regions during the routing
- Opportunistic routing for minimum EC is performed
- Binary tree concept is implemented to avoid the packet collision
- Efficient route selection with least number of active nodes, minimum RPER and affordable delay

The rest of the paper proceeds as follows: Section 2 covers the literature review of the state-of-the-art work and system model of proposed protocols is presented in Section 3. Simulation results, feasible regions and performance evaluation parameters of proposed protocols are explained in Section 4. Finally, Section 5 presents the conclusion and future work of the paper.

## 2. Literature Review

**Figure 1:**
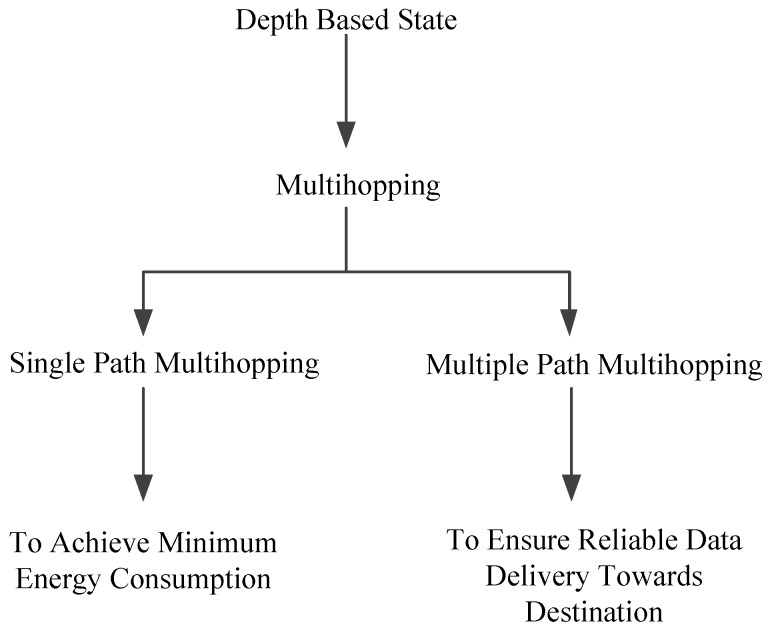
Pictorial Description of Depth-Based Protocols.

In this section, the existing protocols are discussed, which are energy efficient and achieve data reliability with maximum throughput. The state-of-the-art depth-based routing protocols are categorized into two types: multi-hopping via single path and multi-hoping via multiple paths. Single path concept is used for minimum EC to avoid void hole regions. In contrast, multi-path routing helps to ensure reliable data delivery towards the destination. Pictorial form of the aforementioned literature is presented in Figure 1.

The author proposed the Geographic and Opportunistic Routing with Depth Adjustment (GEDAR) in UWSNs in [9]. This protocol performed anycast geographic opportunistic routing. Therefore, the selection of next potential neighbors is anycast. Recovery mode is active in proposed protocol to manage the void hole region and to move the nodes into a new location after depth adjustment recovery method. GEDAR efficiently solved the problem of void hole region. It also increased the PDR through depth adjustment mechanism. However, position adjustment mechanism consumed much energy and increased end-to-end delay in the data packet delivery.

The routing protocol HYDROCAST is proposed in [10] to route the data packets towards destination by measuring pressure level information by Lee et al. Anycast mechanism can be followed to route the data packets in only vertical direction. Data from a single source is forwarded to a subset of forwarders using a greedy approach. The receiver does not forward the data packets until all forwarder nodes fail. Data is transferred to the higher depth nodes when there exists a possibility of the data to enter in void hole regions. In this case, data is directed towards sink using different paths. Protocol’s reliability is enhanced by increasing transferring DR towards forwarders through different paths. This mechanism helped to avoid void hole regions in the network. However, HYDROCAST protocol must bear the cost of both energy and end-to-end delay simultaneously.

Another Layered Multi-path Routing Control (LMPC) protocol is proposed in [11] by Junfeng et al. LMPC sends many copies of data packets towards a destination through different routes. To achieve the reliability in data binary tree generation concept is introduced in the proposed protocol. Generation of a binary tree from the source node to destination enhanced the EC. However, sparse area generation with randomly deployed nodes result in packet drop and the unsensed zone is created in the network.

Ali et al. proposed two routing protocols in [12] which are depth-based and known as: FLMPC-One and FLMPC-Two. Both proposed protocols followed the positive aspect of LMPC. The features which differentiate FLMPC from LMPC is that FLMPC generates a binary tree from sensor node instead of the source node and cover the void hole regions problem by introducing cross nodes concept. The proposed protocol reduced the number of retransmissions. However, 3-hop neighbors information and generation of binary tree results in high EC.

In [13], Yu et al. proposed a protocol which depends on weighting depth and forwarding area division (WDFAD-DBR) for UWSNs. It is done to increase the reliability of data by decreasing the probability of data packets to enter in void hole regions. The authors measured the sum of depth differences from the source node to next expected hop node to avoid void hole regions. Theoretical analysis is performed in this article using channel contention. Energy efficacy and data reliability are successfully achieved from the proposed protocol. However, sparse regions effect Packet Delivery Ratio (PDR) with the propagation and processing time at every hop node.

In [14], Geographic Forwarding based on Geo-spatial and Greedy Forwarding Division (GFGD) and Greedy Geographic Forwarding based on Geo-spatial Division (GGFGD) protocols are proposed by Jiang et al. GFGD is a geographic multi-path routing protocol based on geo-spatial division in duty-cycled UWSNs. In this work, all sensor nodes can switch their states from active to sleep and vice versa which can save EC of the network. A node with shortest path loss and highest energy with minimum delay is selected as next forwarder in both GFGD and GGFGD. Greedy forwarding approach is used in both protocols. GFGD takes directional forwarding into account and saves energy with affordable delay as compared to GGFGD. Next selection of neighbors in GGFGD increase the delay in processing and propagation.

Latif et al. proposed Spherical Hole Repair Technique (SHORT) protocol in UWSNs [15]. SHORT is a spherical repair technique. This protocol avoids energy holes and coverage holes. In this protocol, if a node is about to die, it broadcasts a control message to its neighbors. Therefore, neighbors stop forwarding the packets to this node. To fill this coverage, a sensor node from the dense region is introduced into this region by ensuring no coverage hole in another location in the network. The nodes with hidden cross triangles have a higher probability to move into a new position removing the coverage hole. This mechanism increases the throughput and lifetime of the network at the cost of high end-to-end delay.

In [16], Reliable Energy-Efficient Pressure-Based Routing (RE-PBR) protocol is proposed by Khasawneh et al. RE-PBR is reliable and energy efficient pressure-based routing protocol. Link eminence, depth and the enduring energy constraints are taken into account for the selection of forwarder nodes towards the destination. This scheme successfully achieved the balanced EC due to the avoidance of immutable forwarder-selection of neighbors.

OR protocol is proposed for void hole region avoidances in [17]. In this routing protocol, instead of the current void node retrieval method, depth adjustment technique is used to change the position of nodes in the vertical direction. Adjacency graph of neighbor nodes is maintained at every node to select the potential-forwarder nodes towards the destination. This protocol can bypass the void nodes by using directional routing in every direction. However, the compensation of high EC persists in [17].

In this paper, Ahmad et al. proposed Geo-Opportunistic Routing (GOR) protocol of immutable selection of forwarder node to balance the EC and void hole regions in UWSNs [18]. The total volume of the network is distributed into cubes to reduce the intrusion problem and to make well-versed decisions for proficient energy use. Moreover, data packets are recovered from the void regions by introducing mobile sinks to reduce the traffic flow. However, sparse regions effect the propagation time in proposed protocols.

Three Efficient routing protocols named: Sparsity-aware Energy-Efficient Clustering (SEEC), Circular Sparsity-aware Energy-Efficient Clustering (CSEEC) and Circular Depth-based Sparsity-aware Energy-Efficient Clustering (CDSEEC) are proposed in [19] by Sher et al. Proposed protocols helped to monitor the fields with square and circular geometries in UWSNs. The purpose of the proposed work is to minimize the energy in sparse regions and to stop the redundant transmissions. Therefore, the increase in the sparse region also increases the energy and communication overhead. As, energy dissipation has direct relation with distance. Therefore, as the sparse region increases, energy overhead also increases.

Energy-Efficient Multi-hopping Routing (EAMR) protocol is proposed by Cengiz et al. in [20]. Fixed clustering is performed in the proposed protocol. EAMR successfully minimized the overhead in communication with which lifetime of the network is also increased. Inter-cluster transmission increases the network scalability. However, in sparse regions cluster heads cannot bear load [20].

The authors in [9,10,11,12,13,14,15,16,17,18,19,20] have achieved specific parameters, i.e., minimum EC, duplicate packets generations and void hole avoidance in UWSNs. In LMPC [11], the source node established the binary tree; while in [12], every cross node established the binary tree. This distribution of nodes in [11,12] resulted in duplicate packet generation and delay with high EC during the packets transmission. However, these protocols do not provide a feasible solution due to high EC and PRR along with duplicate packets generation. To tackle the aforementioned problems, positive features of both FLMPC-one and FLMPC-two are exploited, and two novel proficient protocols are proposed by considering real-time performance parameters, i.e., delay, EC, PRR and RPER. Towards efficient energy use, efficient route selection is taken into consideration and proposed protocols are exploited to achieve the minimum EC and RPER with affordable delay. Proposed protocols outperformed in void holes avoidance by giving minimum RPER as compared to the existing protocols. Table 3 summarizes the literature review.

### 2.1. Summarized Literature Review

The focus of the researchers is on EC minimization through different routing protocols. All protocols are designed for different objectives according to the scenario. Depth-based routing uses single path which emphasis on energy efficacy by overlooking void hole regions and other network procedures. In multi-path routing protocols, the focus is on reliability of data from source to destination. EC is not taken into account in this scenario. Void hole avoidance considered both parameters simultaneously: energy use and data reliability. However, some compromises in terms of latency and EC also need to be faced because of void nodes origination which lead the network towards maximum EC and degrades the network lifetime.

### 2.2. Uniqueness of Proposed Protocol from Existing Protocols

In this paper, similar to FLMPC, two routing protocols are proposed. However, they are different from existing as follows:

- In SPF-Three, the single shortest path for routing of data packets based on ’Dijkstra’ algorithm
- In BFS-SPF-Three, breadth First search for routing and opportunistic routing for minimum EC
- Efficient routing selection with the least number of active nodes in both SPF-Three and BFS-SPF-Three
- Above discussed strategies provide results in the reliability of data transmission with minimum EC and least distance

## 3. System Model and Description

In this section, firstly optimal structure for shortest path selection and working of ’Dijkstra’ algorithm is discussed. Then, the system models of both the existing and the proposed schemes are demonstrated. FLMPC-One and FLMPC-Two are generalized as FLMPC to elaborate the model of existing protocols. Then the propagation of the underwater acoustic signal is discussed. Later, two protocols SPF-Three and BFS-SPF-Three are proposed to make routing decisions based on ’Dijkstra and Breadth first shortest path-based’ algorithm with 3-hop neighbors information. Reliability in transmission and entrance of data packets in void hole regions is eliminated. Moreover, proposed schemes efficiently reduce the EC by making routing decisions (based on a greedy approach). In next subsections, optimal structure for shortest path selection, ‘Dijkstra Algorithm’ and system model of both existing and proposed protocols are discussed.

### 3.1. Optimal Structure for Shortest Path Selection

The property of shortest path selection is explained as: if P(i,j) = $\left(V_{i}\ldots V_{k}..V_{s}\ldots V_{j}\right)$ is the shortest path from node *i* to the node *j*. Node *k* and *s* are the intermediate relay nodes then P(k,s) must be the shortest path from *k* to *s*. Suppose, $P\left(i,j\right)=\left(V_{i}\ldots V_{k}..V_{s}\ldots V_{j}\right)$ is the shortest path from node *i* to node *j*, then P(i,j)=(P(i,k)+P(k,s)+P(s,j)). If $P^{\prime }\left(i,k\right)$ is the shortest distance from node *i* to node *k*, then there must be a shortest path $P^{\prime }\left(i,k\right)$ from node *i* to node *k* and $P\left(i,j\right)=(P^{\prime }\left(i,k\right)+P\left(k,s\right)+P\left(s,j\right))\leq P\left(i,j\right)$. Meanwhile, P(i,j) is the given shortest path from node *i* to node *j* in contradiction. The P(k,s) is the shortest distance from node *k* to node *s* and P(k,j) is the shortest distance from node *k* to *j*.

### 3.2. ’Dijkstra’ Algorithm

If there is a shortest path $\left(V_{i},V_{k},V_{j}\right)$ from node *i* to *k* and $V_{i}$ is the vertex in front of the $V_{k}$, then $\left(V_{i}\ldots V_{k}\right)$ must be the shortest path from *i* to *k*. ’Dijkstra’ algorithm finds the global shortest path choosing local best shortest path among the adjacent vertexes.

### 3.3. System Models of Existing and Proposed Protocols

To explain the system models of existing and proposed protocols, a few key terms are defined here: the nodes near or at the layers are known as Cross nodes. The nodes that sense their own data before forwarding the data packets towards higher depth nodes are known as Normal nodes (relay nodes).

The network consists of cross, normal, source and sink (sonobuoy) nodes with the surface gateways and channel pathways. These sonobuoys act as an embedded system in underwater environment. In the proposed protocols, relay nodes are randomly deployed for data transmission in the deep ocean by targeting minimum EC. Initially, the relay nodes are in a sleeping mode with minimum EC. However, they become active on receiving the data packets from the source and cross nodes. When these relay nodes complete their participation in communication, they sleep again. The binary tree formation is the main feature of both proposed schemes with some additional properties of SPF-Three. This approach finds the single shortest path towards the destination (based on ”Dijkstra and BFS-SPF-Three” algorithm) which searches different possible shortest paths and performs opportunistic routing to minimize the EC and distance.

**Figure 2:**
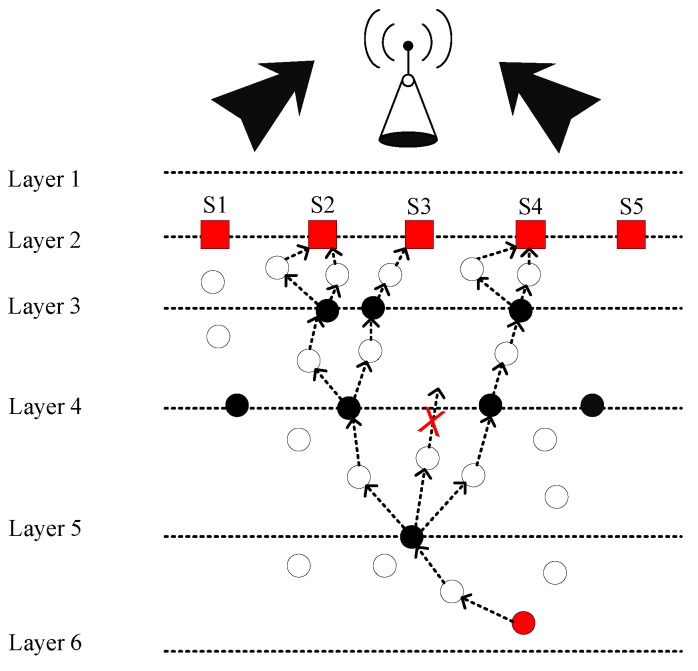
System Model of FLMPC-Two.

**Figure 3:**
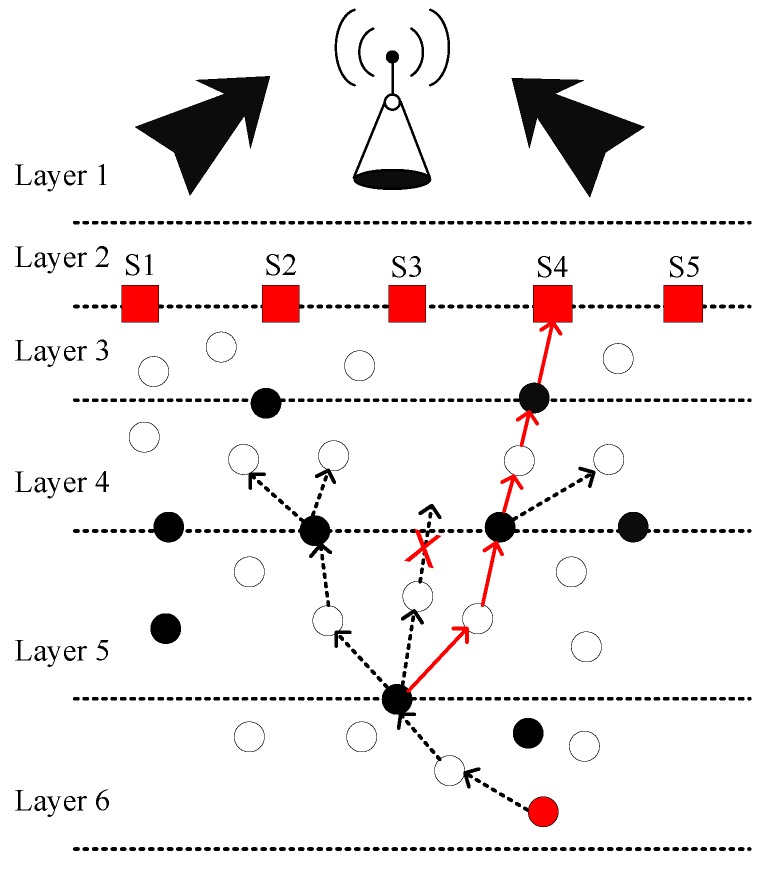
System Model of SPF-Three.

**Figure 4:**
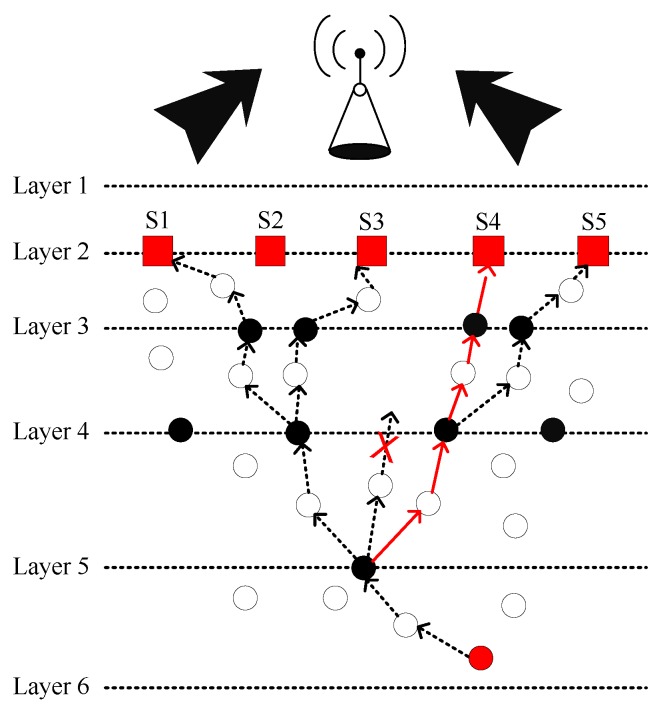
System model of BFS-SPF-Three.

**Figure 5:**
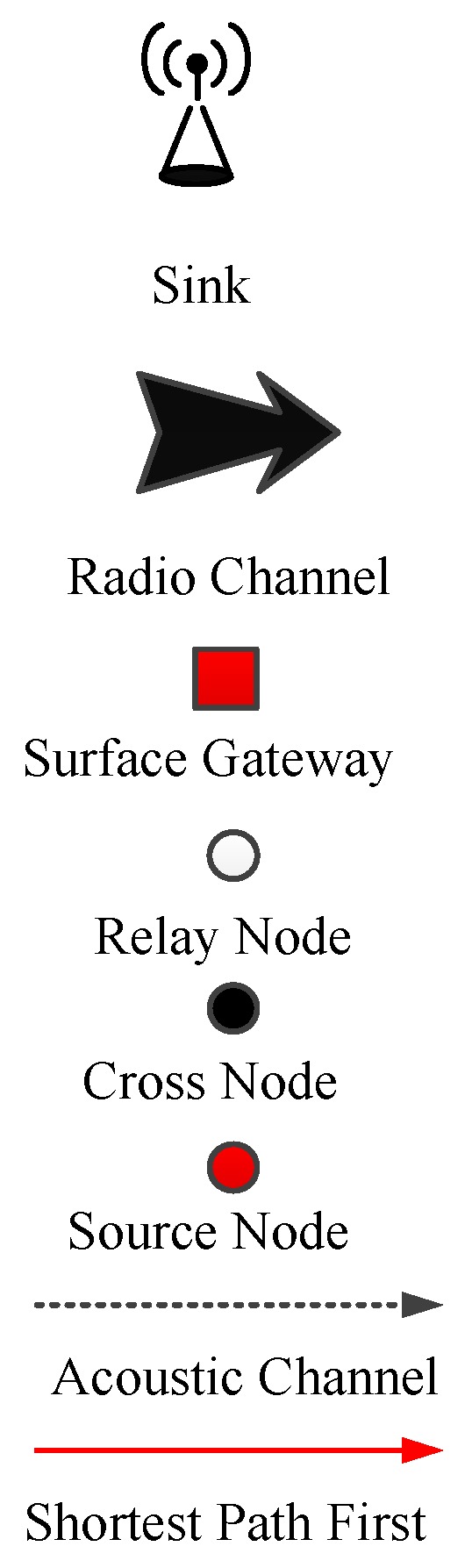
Legends of above Discussed Protocols.

Figure 2 demonstrates the system model of FLMPC protocol with non-homogenously distributed layers to overcome the noise hindrance. In the existing schemes, every cross node keeps two copies of data and forwards the data using IP-based broadcasting. The main aim is to balance the effect of collision till the packet reached the destined sonobuoy. Distribution of layers depends on different types of noises in the sea depth, i.e., in shallow water noise of ships is high as compared to sea depth. Therefore, in shallow water layers will be closer to each other. System models of both proposed schemes are shown in Figure 3 and Figure 4. The Figure 5 demonstrates legends for the aforementioned proposed and existing protocols. In proposed protocols, there is a need to find the path which is the shortest path with the minimum EC and distance. Due to the ability of shortest path selection, SPF-Three and BFS-SPF-Three become more efficient. In this regard, network communication space is divided into unequal multiple layers and becomes narrow near the sea surface. To handle the effects of packet collision and data loss, binary tree generation idea is adopted from the existing protocols with 3-hop neighbors information. By considering the aforementioned issues, two efficient routing protocols are proposed. The detailed description of the routing protocols is discussed below.

#### 3.3.1. Forwarder Model

Propagation idea of the proposed protocols and acoustic signals with their absorption constraints in UWSN environment are discussed below.

##### Channel Fading

**Figure 6:**
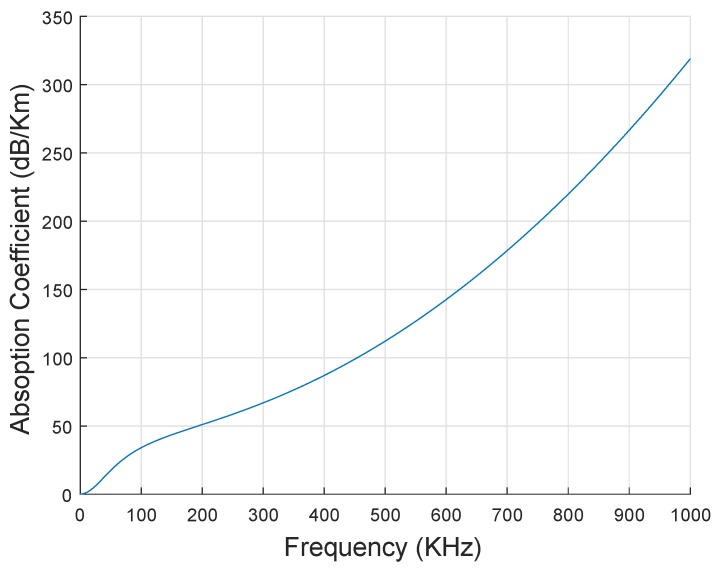
Absorption and Frequency Relation.

The sudden change in signal strength over a distance ‘*D*’ and frequency ‘*F*’ is calculated using Equation (1) [21].

(1) $$\;A\left(D,F\right)=D^{k}\times \alpha \left(F^{D}\right),$$

here, ‘α(F)’ is the absorption coefficient, while signal range define the signal geometric propagation which may be cylindrical, practical or spherical. In [13], the authors define ‘α’ using Equation (2).

(2) $$\;\alpha \left(F^{D}\right)=\frac{0.11F^{2}}{1+F^{2}}+\frac{44F^{2}}{4100+F^{2}}+2.75\times 10^{-4}F^{2}+0.003,$$

here, ‘F’ is measured in KHz and ‘α(F)’ is measured in dB/Km, respectively. In sea water, different noise resources (shipping noise, thermal noise and turbulence noise) exits and denoted by ‘NR’. For the given frequency ‘F’, the overall noise power denoted by ‘NP’ with spectral density is calculated using Equation (3) [22]. Figure 6 shows the relationship of ‘$\alpha {\left(F\right)}^{\prime }$ and ‘F’ using Thorp’s model. According to Thorp’s model, absorption of acoustic signals grows with the growth of ‘F’.

(3) $$\;NP\left(F\right)=\sum_{i=1}^{NR}NP_{i}\left(F\right),$$

‘$NP_{i(F)}$’ is measured in dB and it should be in between (1-NR). This attenuation relates directly with the distance of source from destination. This attenuation over ‘D’ is calculated using Equation (4).

(4) $$\;\;NP_{i}^{D}\left(F\right)=\sum_{i=1}^{NR}\frac{NP_{i}\left(F\right)}{D^{k}\alpha \left(F^{D}\right)}.$$

##### Channel Capacity

Channel capacity is denoted by ‘C’ and it is the number of bits without error using bandwidth ‘B’. The signal to noise ratio ‘SNR’ and the actual capacity is calculated using Equations (5) and (6). Whereas, packet error rate is denoted by ‘PER’.

(5) $$\;C=B.\log_{2}\left(1+SNR\right),$$

(6) $$\;Actual\;Capacity\left(PER\right)=\frac{C}{1-H_{2}\left(PER\right)},$$

here, ‘$H_{2}$’ is a binary entropy function using PER, which is defined as $H_{2}=\left(PER-1\right)\log_{2}\left(1-PER\right)\left(PER\log_{2}PER\right)$.

##### Transmission and Receiving Energy

‘$E_{t}$’ represents the transmission energy and ‘$E_{r}$’ is used to denote receiving energy. Equation (7) is used to calculate ‘$E_{t}$’ and Equation (8) is calculating ‘$E_{r}$’. Whereas, transmission power is denoted as ‘$P_{t}$’, reception power as ‘$P_{r}$’, distance from *n* − 1 to *n*th node as *D* and data rate as ‘DR’, respectively. Where, ‘*N*’ is the total number of data packets ‘DP’ and ‘THCs’ are represents the total hop counts. In addition, packet length is denoted as ‘PL’, total leftover energy of the network nodes as ‘$E_{t}^{remaining}$’ and total receiving energy of the network nodes after receiving the data packets as ‘$E_{r}^{remaining}$’. The ‘$E_{t}^{remaining}$’ and ‘$E_{r}^{remaining}$’ are calculated using Equations (9) and (10).

(7) $$\;E_{t}=\frac{P_{t}\times PL}{DR}\times D,$$

(8) $$\;E_{r}=\frac{P_{r}\times PL}{DR}\times D,$$

(9) $$\;E_{t}^{remaining}=\sum_{DP=1}^{N}\sum_{HC=1}^{THC}n_{i}-E_{t},$$

(10) $$\;E_{r}^{remaining}=\sum_{DP=1}^{N}\sum_{HC=1}^{THC}n_{i}-E_{r}.$$

Initial energy is represented as ‘$E_{Initial}$’ and total energy of the network as ‘$E_{Total}$’. Total energy is calculated using Equation (11).

(11) $$\;E_{Total}=E_{Initilial}-\left(E_{t}^{remaining}+E_{r}^{remaining}\right).$$

#### 3.3.2. Layering

**Figure 7:**
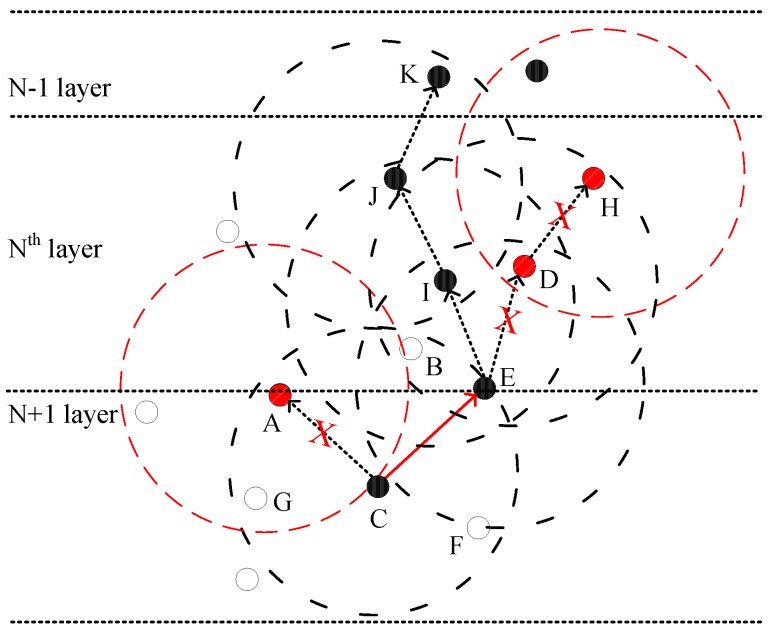
Forwarder Node Selection in SPF-Three and BFS-SPF-Three Layered Multi-path Routing Protocol.

**Figure 8:**
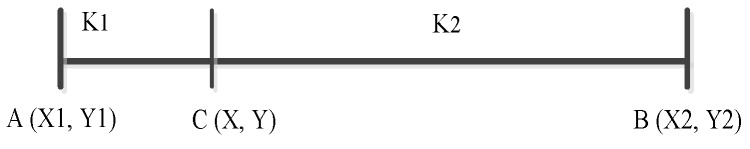
Layer Division.

In the proposed schemes, network is divided into unequal layers (see Figure 7) due to high noise rate at sea surface as compared to sea depth. Therefore, layers are closer to each other as the data packets move near the surface. This happens to minimize the effect of noises in UWSN environment. The basic purpose of the proposed approach is to increase the reliability of the data packet and minimize the ratio of packet drop. The expression for the unequal size of layers distribution is given in Equation (12).

(12) $$\;C\left(x,y\right)=\frac{k_{1}x_{2}+k_{2}x_{1}}{K_{1}+k_{2}},\frac{k_{1}y_{2}+k_{2}y_{1}}{K_{1}+k_{2}},$$

here, the endpoint coordinates are represented by $x_{1},y_{1},x_{2}$ and $y_{2}$. Whereas, C(x,y) is the point of division as shown in Figure 8. In this figure, point *C* divides the line in $K_{1}$:$K_{2}$ that depends on the strength of noise. Cross nodes are uniformly distributed, so every packet passes through them without leaving any space.

#### 3.3.3. Neighbors Selection

In the proposed protocols, neighbors list is maintained by every node in the transmission range. They broadcast the message in the network. This message includes the source node, destination node, location, and depth ’yd’ of the source node. Receiving node calculates the distance from the source to the destination node using Euclidean distance which is expressed as Equation (13);

(13) $$\;Distance_{\left(i,j\right)}=\sqrt{{\left(x_{1}-x_{2}\right)}^{2}+{\left(y_{1}-y_{2}\right)}^{2}},$$

where $\left(x_{1},x_{2}\right)$ and $\left(y_{1},y_{2}\right)$ are the coordinates of source and receiver node. Every sensor node maintains a table of their source ID, location, depth, and distance from each neighbor. Coordinates (x,y) are obtained from the control message, while distance is calculated using Equation (13) and the depth ’yd’ is obtained by calculating the depth of the node from the sea surface. After getting the required tuple, following constraints help the proposed protocols to find the optimum neighbor as forwarders.

- $Distance_{\left(i,j\right)}\leq TransmissionRange\left(R_{t}\right)$
- $Depth_{i}>Depth_{j}$

This depth of sensor nodes helps to find the current forwarder which is closer to the sea surface. Information regarding higher depth node is not required in the proposed protocols to eliminate the extra EC and communication overhead.

### 3.4. Network Configuration and Data Transmission

In this phase, SPF-Three finds the potential-forwarder neighbors from the neighboring table with the shortest path. BFS-SPF-Three performs the opportunistic routing to find the best shortest path with minimum energy overhead. The schemes with their algorithms are discussed in detail below:

#### 3.4.1. SPF-Three

SPF-Three forwards the data packets after ensuring the information of next 3-hop neighbors from the current node to avoid the void regions. The shortest path is obtained from the proposed protocol based on ’Dijkstra’ algorithm. It selects the best route for the underwater communication. Algorithm 1 demonstrates the selection and transmission of SPF-Three.

#### 3.4.2. BFS-SPF-Three with Opportunistic Routing

BFS-SPF-Three also forwards the data packets same as SPF-Three after ensuring the information of next 3-hop neighbors from the current node to avoid the void regions. BFS-SPF provides shortest paths among all available paths then BFS-SPF-Three performs opportunistic routing. It selects the single shortest path with minimum EC and least distance from the sea surface. Selection and transmission of BFS-SPF-Three is presented in Algorithm 2.

**Algorithm 1** SPF-Three Pseudocode.

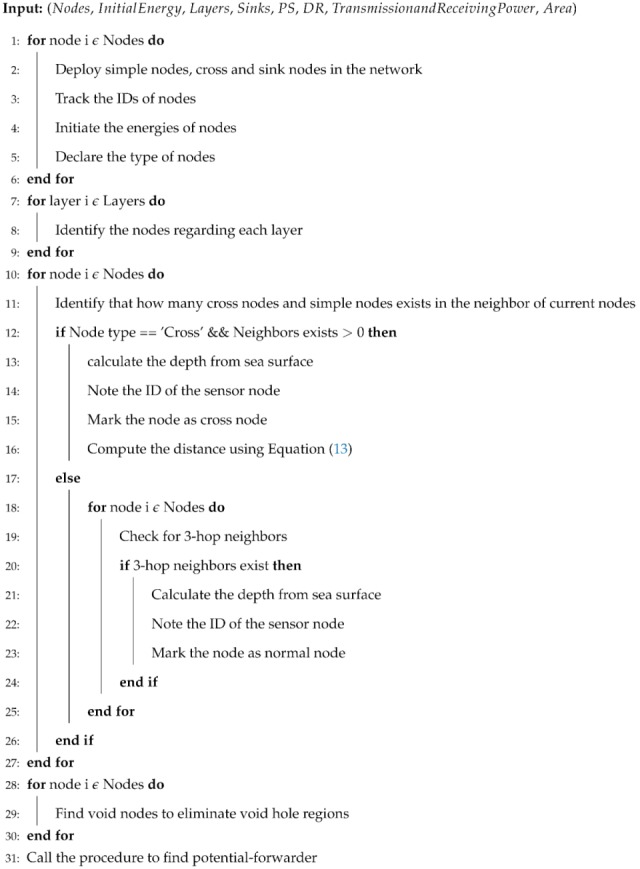


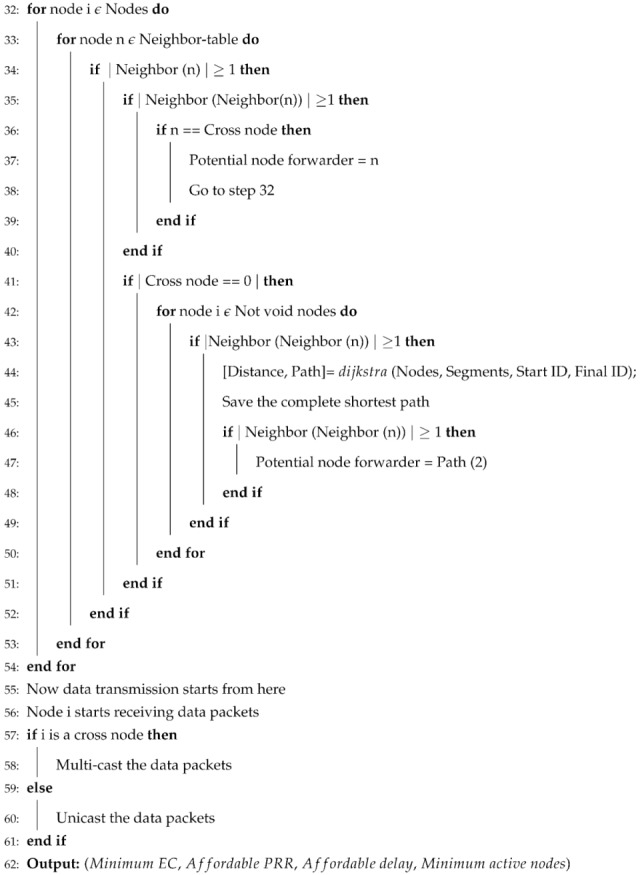



**Algorithm 2** BFS-SPF-Three Pseudocode.

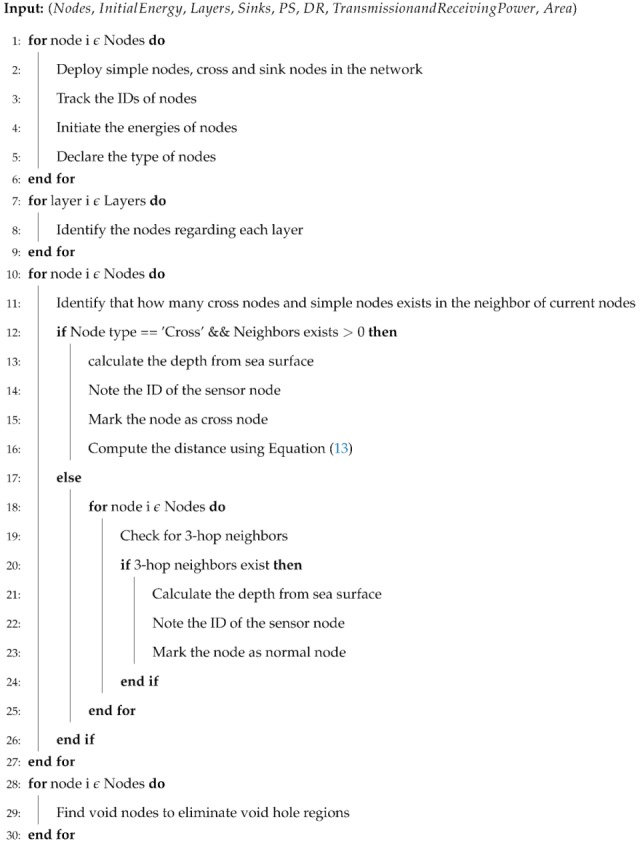


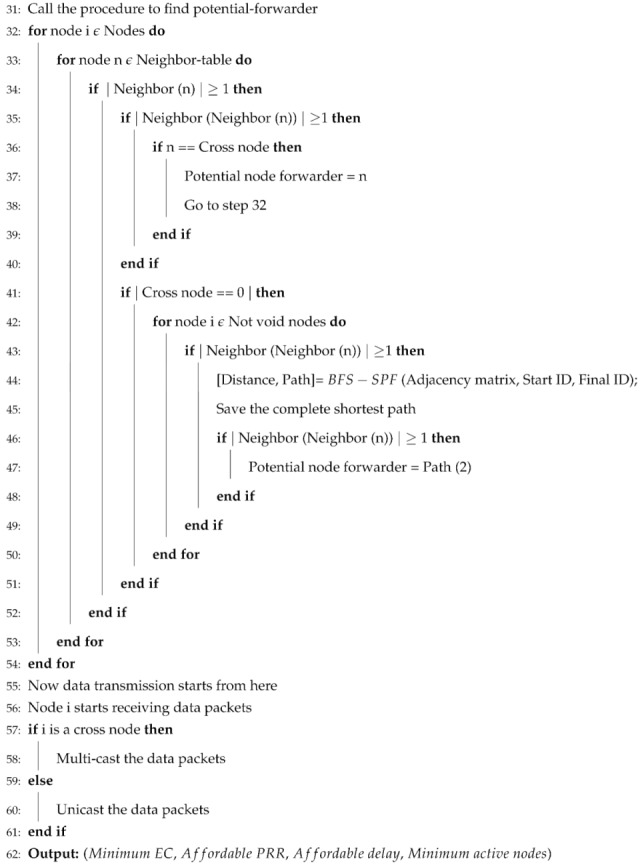



### 3.5. Binary Tree Generation

Following two conditions must be true for transmission of data packet from a node.

- A sensor or relay node must be used for linear transmission
- There must be a cross node for binary tree.

If the node is a simple sensor node it will unicast the signal. Otherwise, binary tree formation starts with a source node acting as a root and neighbor nodes as leaves. Each parent node has almost two child nodes. In the proposed protocols, the main characteristics used in the generation of a tree is cross node.

## 4. Simulations Results and Discussion with Performance Evaluation

In this section, the performance of the proposed protocols is evaluated, i.e., SPF-Three and BFS-SPF-Three. Furthermore, the comparative analysis of existing and the proposed protocols is presented. In both proposed and existing protocols, the packets are forwarded from source node towards sink (sonobuoys on the surface of the sea in a greedy manner). The main difference is that the existing protocols only sense the next 2-hop neighbors or 3-hop neighbors to find the best potential-forwarder (with a low probability of avoiding void hole regions). Whereas, the proposed protocols sense the next 3-hop neighbors to find the best potential-forwarder. Meanwhile, choose the best shortest path from that forwarder towards the destined sonobuoy by completely avoiding the void hole regions. This shortest path selection results in minimum delay with minimum EC. The proposed protocols eliminate the chance of packet loss in the void holes. In the next phase, the experimental setup of implemented protocols is discussed.

### 4.1. Simulations Setup

In simulations, two different scenarios of FLMPC protocols are implemented. In both scenarios, 150 nodes are deployed in (2000 × 2000) m${}^{2}$ area. Extensive simulations are done to verify the compatibility of the proposed protocols. The performance parameters and network dimensions for the simulations are listed in Table 4.

### 4.2. Performance Metrics

In this section, the proposed and existing protocols are evaluated based on these defined terms.

***Active nodes*** represent the nodes which are taking part in communication.

***Delay*** is defined as the time taken by the packets to reach the destination. It includes the following delays in transmission:

- Delay in data packet transmission
- Delay in data propagation
- Delay of packet holding
- Delay in data Processing

In the present scenario, multiple surface gateways for multiple data packets are destined. These data packets pass from different gateways and experience delay. However, the highest delay among these delays is considered in the current scenarios.

***EC*** is the amount of energy used by the nodes during whole communication in UWSNs. Total EC is the accumulation of all sensor nodes energy that takes part in communication.

***RPER*** is the number of erroneous bits allowed in data packets to be received at the destination. Surface gateways or sonobuoys drop the data packets if erroneous bits exceed the RPER limit.

***PRR*** is the ratio of successfully received packets at destination sink.

### 4.3. Simulation Results

In this section, a comparative analysis of proposed protocols and existing protocols are discussed. Several active nodes per layer, EC, delay, RPER and PRR are considered verify the performance of the proposed protocols. The detail of each performance parameter is presented as follows:

#### 4.3.1. Active Nodes per Layer

**Figure 9:**
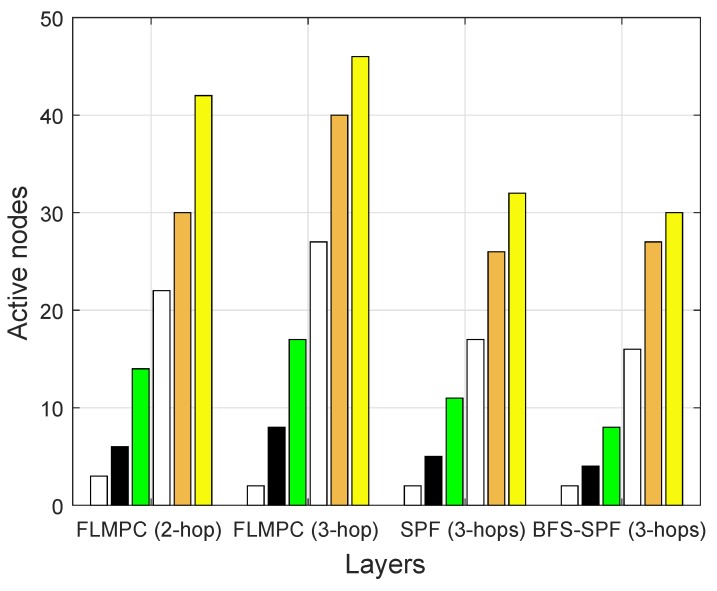
Number of Active Nodes per Layer.

Several active nodes are an important parameter that effect the network lifetime. The most important key parameter used to prolong the network lifetime is the number of active nodes. Figure 9 shows the number of active nodes per layer for both proposed and existing protocols. In existing protocols, the number of active nodes increases with the increase in number of layers. Because if the source node lies near the surface gateway, it results in fewer active nodes and awakes fewer nodes. In both FLMPC-One and FLMPC-Two, multiple routes are originated from every cross node to form a binary tree for reliable data delivery. This binary tree generation actives a large number of nodes. In the FLMPC-One, active nodes are more than the FLMPC-Two. The number of active nodes increases, while moving from lower to the upper layer of the underwater network to forward the data packets. The main reason for the increase in the number of active nodes are the cross nodes. The cross nodes originate multiple copies of data packets and generate a binary tree for reliable data delivery. In FLMPC-Two, the number of active nodes are less than the FLMPC-One because in FLMPC-One, a source node looks forward up to 2-hop which leads the data packet in void region. Meanwhile, FLMPC-Two looks forward up to 3-hop and reduces the probability of packet delivery in void hole region. The aforementioned idea minimizes retransmissions and the number of active nodes.

In contrast, the SPF-Three finds the shortest path to reach the destination by avoiding void hole regions completely. Therefore, active nodes in SPF-Three are less than the existing protocols. Meanwhile, the BFS-SPF-Three finds the different possible shortest paths in breadth wise and performs opportunistic routing by selecting the best potential-forwarder nodes. The aforementioned route selection mechanism efficiently minimized the EC. It contains the fewer active nodes and minimum distance. The number of active nodes in each scheme is shown in Figure 9. Figure 9 clearly depicts that active nodes in BFS-SPF-Three are lesser than SPF-Three and the aforementioned existing protocols. Both proposed protocols outperformed. It makes smart decisions for the reliable route selection. Therefore, suitable nodes are selected for communication to prolong the network lifetime.

#### 4.3.2. Packets Delay

**Figure 10:**
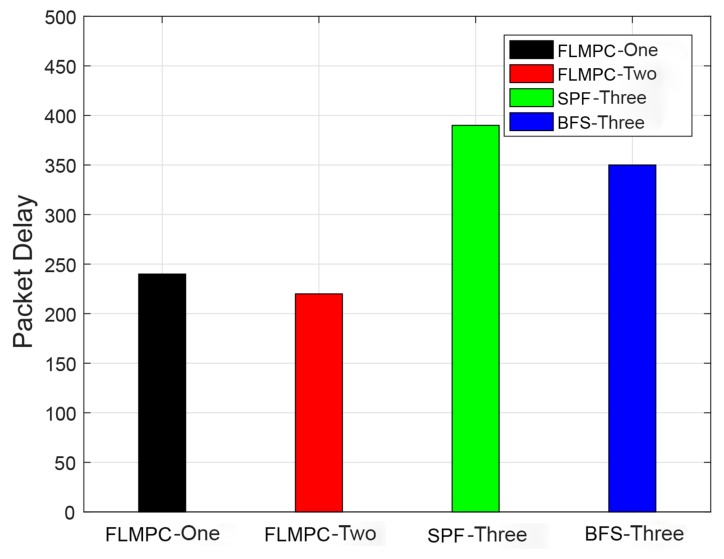
Packet Delay (ms).

Delay is the most important parameter to be considered in UWSNs for communication because sometimes a small delay in a network collapse the entire system. Delay in packets is mainly influenced by the channel used in UWSNs and the distance from the source to destination. Therefore, to tackle this important problem, two routing protocols are proposed. Delay of the previously discussed protocols is shown in Figure 10. Delay in FLMPC-One is higher than FLMPC-Two because FLMPC-One 2-hop neighbors information leads to a high number of active nodes. Meanwhile, delay of SPF-Three is greater than all aforementioned protocols. The reason behind this delay is that SPF-Three finds the shortest path from source to destination. It collects 3-hop information as FLMPC-Two, which results in the unique path with unintentionally packets collision. Due to the above-mentioned reasons, the number of data packets are unable to reach the destination because of massive collisions( the ratio of packet drop is high). Therefore, SPF-Three retransmits the data packets using the shortest route with minimum EC. However, they exhibit delay but provides reliable data delivery. In BFS-SPF-Three, the delay is less than the SPF-Three and higher than existing protocols because of binary tree generation. In addition, BFS-SPF-Three returns all shortest possible routes from source to the destination by exploiting the binary tree breadth wise and by performing opportunistic routing. Opportunistic routing in BFS-SPF-Three selects the route with minimum distance and provides reliable packet delivery. The farthest nodes selection causes the delay in packets delivery and enhances the propagation time.

It is evident that as the layers increases, the active nodes increase which results in an end-to-end delay. At layer 6, the minimum delay is 0.82 s and 0.63 s in BFS-SPF-Three. In existing protocols, a total end-to-end delay is 240.6178 ms and 220.1483 ms while in proposed protocols end-to-end delay is 390.4561 ms and 351.0972 ms.

#### 4.3.3. EC

**Figure 11:**
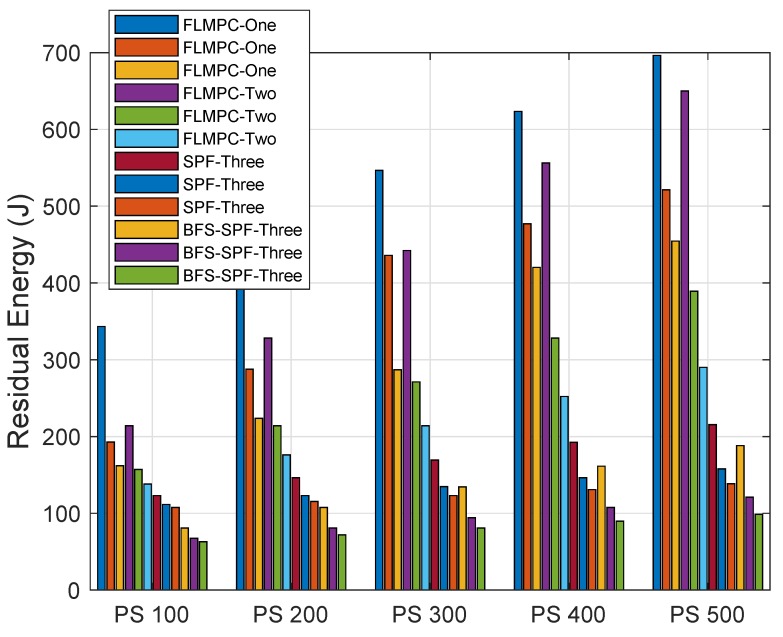
EC with Different PSs and DRs.

The EC of both proposed and existing protocols is shown in Figure 11 with different PSs and DRs. A DRs of 10, 20 and 30 Kbps is considered. PSs is considered to be 100, 200, 300, 400 and 500 bytes. Figure 11 demonstrates that EC mainly depends on DRs irrespective of the packet length. EC increases as the PS increases.

In FLMPC-One and FLMPC-Two, the number of active nodes are higher than the proposed protocols because of binary tree generation from cross nodes. A high number of active nodes create a dense environment which results in duplicate packets generation. Due to collisions in packets, maximum energy is dissipated in retransmissions. The EC increases with the increase in packet size as shown in Figure 11. In both existing protocols, the node performs calculations up to 2–3 hop to avoid the void hole regions. However, the EC is increased for collecting 3-hop neighbors information.

In SPF-Three and BFS-SPF-Three, the EC is less than both existing protocols. However, SPF-Three finds the shortest route towards a destination with fewer active nodes. Meanwhile, BFS-SPF-Three performs the opportunistic routing and finds the shortest route. Binary tree formation and elimination of void nodes are the common characteristics of all protocols. In contrast to existing protocols, the number of active nodes in proposed protocols is less which results in less EC. If retransmissions in proposed protocols become necessary, SPF and BFS-SPF help to find the shortest path with minimum distance and EC. The elimination of void region problem with the best route decision makes the proposed protocols unique. Continuous transmission, reception and the noise of wind and ships also effect the data packets and EC. The proposed protocols outperform regarding minimum EC and tackle the challenges in efficient way to keep the communication continuous. The EC values with different PSs and DRs are shown in Table 5, Table 6, Table 7, Table 8 and Table 9.

#### 4.3.4. PRR

**Figure 12:**
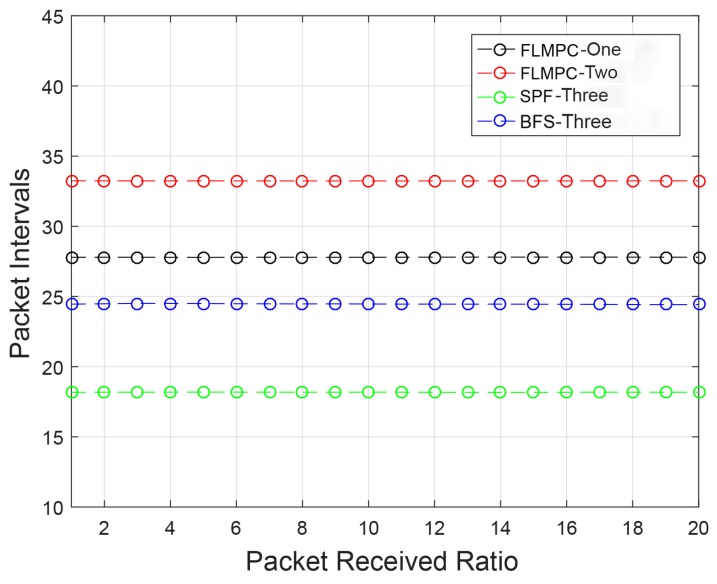
PRR of All Protocols.

The PRR of the existing protocols is high than the proposed protocols as shown in Figure 12. In FLMPC-One and FLMPC-Two protocols, PRR is higher because of the formation of binary trees from cross nodes towards the destined sink. In proposed protocols, the possibility of void nodes is eliminated. The PRR of SPF-Three and BFS-SPF-Three is lower than existing protocols because it selects a single shortest path from the source towards the destination. Opportunistic routing causes a collision in several data packets because of same shortest path selection. This selection results in several packets drop. However, BFS-SPF-Three show high performance as compared to the SPF-Three.

#### 4.3.5. RPER

The EC per packet in the presence of different packet errors is discussed in this section. If RPER is high, the reliability of data and EC of packets transmission increases. When the packets are received at sink the error rate is calculated. If the error exceeds its error limit then this packet is dropped by the protocols. Increase in packet error means that the reliability of data packet decreases, accordingly.

**Figure 13:**
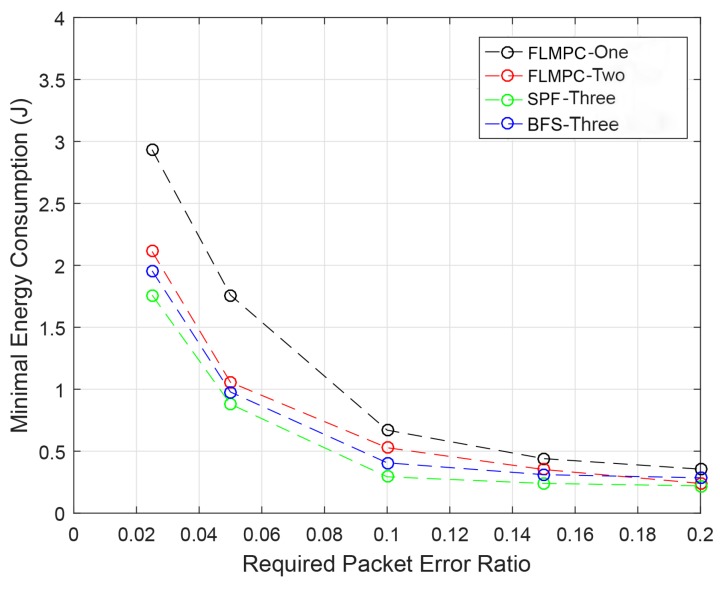
RPER.

In Figure 13, the RPER of all protocols is demonstrated. It is clear from the figure that the EC of packets with different PERs of existing protocols is high than the proposed protocols. Although the difference is minor, because of multiple copies generation in all protocols using binary trees formation. Both proposed protocols select the best route for communication and control the effect of data loss. The proposed protocols are differentiated from the existing protocols because they deal with erroneous bits in a much better way than the existing protocols. Both SPF-Three and BFS-SPF-Three combines the data packets at the sink to demolish the effect of erroneous bits completely.

High messages exchange and void hole formation in existing protocols cause high energy dissipation. Meanwhile in SPF-Three single shortest path reduces the active nodes with a minimum number of messages passing. This selected path decreases the EC by completely removing void hole regions from the underwater network. The proposed protocols deal with void hole regions in an efficient way to achieve the objectives. It is clearly seen from Figure 13, that EC is greater than 0.35 J at 6th second. Hence, the proposed protocols efficiently improved the EC improvements.

### 4.4. Parameters to Optimize

To obtain the best possible results, a mathematical optimization model is adopted. In this subsection, linear constraints are used to formulate the problems. The main objective of the proposed work is to minimize the EC and maximize the throughput. An efficient mechanism is proposed to monitor the constraints of objective functions.

#### 4.4.1. Linear Optimization for Energy Minimization

In SPF-Three and BFS-SPF-Three, the cross nodes broadcast the data packets using IP broadcasting mechanism. However, the relay nodes unicast the data packets. Due to which, minimum EC is required to increase the lifespan of the network. According to the authors in [19], maximum energy is consumed in transmission and reception phase. Nodes consume maximum energy during the active mode and minimum energy during idle mode. However, EC is less as compared to the energy dissipation during transmission and reception. Therefore, the idle node’s EC is not considered. The main objective of EC is expressed in Equation (14) for the total transmission time ($t_{tot}$) to prolong the network lifetime. Main points of Equation (14) are as follows:

(14) $$\;Min\sum_{t=1}^{t_{tot}}\;E\left(t\right),\;\;\;\;\;\forall \;t\;\epsilon \;R.$$

- Energy of sensor nodes is less than and equal to the initialized energy of nodes (because of limited energy resources)
- The DR is less than and equal to DR which make the bits erroneous for transmission. Otherwise, the PER will increase which results in packet drop and packet needs to be retransmitted
- Transmission energy is greater than and equal to energy of the sensor node for transmission
- Link quality should be good for reliable data transmission without error to prevent the packet drop
- Total ‘$E_{t}$’ and ‘$E_{r}$’ is calculated using Equations (15) and (16)
  (15) $$\;Min\sum_{DP=1}^{Nodes}\;E_{t},\;\;\;\;\;\forall \;i\;\epsilon \;R,$$
  (16) $$\;Min\sum_{DP=1}^{Nodes}\;E_{r},\;\;\;\;\;\forall \;i\;\epsilon \;R.$$

#### Graphical Analysis of ‘$E_{t}$’ and ‘$E_{r}$’

Let us consider a scenario in which initial energy of the nodes be 6 J, transmission power of 0.66 mW, PL of 500 bytes, DR of 10 kbps with distance ranges from 100–300 m and the receiving power of 0.395 mW. So,

- 3.3 mJ ≤ $E_{t}$ ≤ 9.9 mJ
- 1.98 mJ ≤ $E_{r}$ ≤ 5.93 mJ

**Figure 14:**
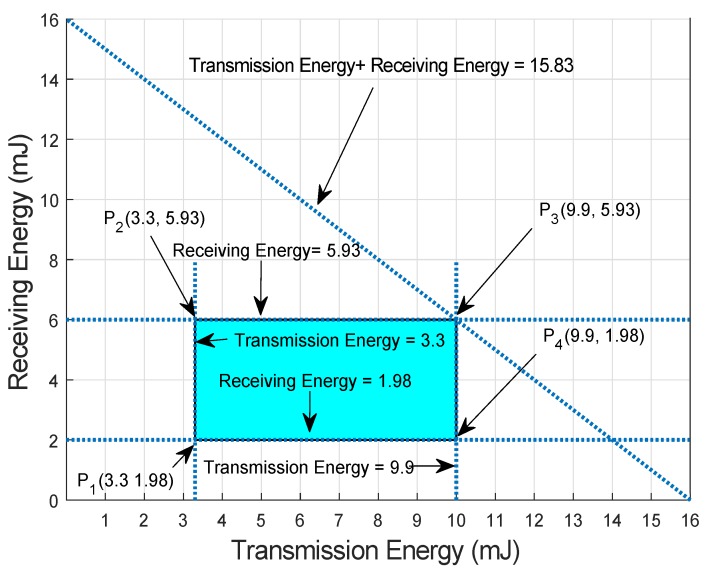
EC Feasible Region for Both Proposed Protocols.

Figure 14 shows feasible region of proposed protocols. Every point in feasible region indicated the feasible solution of the proposed protocol. In vertices of feasible solution, point $P_{1},P_{2},P_{3}$ and $P_{4}\;$ cover the corner points lies within the range of initial energy. Therefore, all the solutions are valid. Every value of energy is taken from this feasible solution while transmitting and receiving of data packets.

### 4.4.2. Linear Optimization for Throughput Maximization

In this subsection, linear programming is used to mathematically formulate the basic objectives of the proposed protocols, i.e., maximum throughput. In the proposed protocols, throughput indicates the number of different packets successfully received at the destined sink. This objective is expressed in Equation (17). Whereas, P(t) is the packet type with counter c in Equation (18):

(17) $$\;Max\sum_{t=1}^{t_{tot}}\;P\left(t\right),\;\;\;\;\;\forall \;t\;\epsilon \;R,$$

(18) $$\;Max\sum_{t=1}^{t_{tot}}\;P\left(t\right)*c;\;\;\;\;\;\forall \;c\;\epsilon \;R,$$

whereas, the value of P(t) is equal to 1, if the packet is unique and P(t)=0 if the packet is duplicate. Main points of Equation (18) are as follows:

- Energy of sensor nodes is less than and equal to the initialized energy of nodes (because of limited energy resources)
- DR is less than and equal to DR which make the bits erroneous for transmission (otherwise PER will increase which results in packet drop and packet needs to be retransmitted)
- Transmission energy is less than and equal to the energy of the sensor node for transmission
- PER should be less than and equal to RPER
- Minimum thermal and shipping noise using Equations (19) and (20)

Thermal and shipping noises are calculated using Equation (19) and Equation (20), respectively. Whereas, $d_{S-G}$ is from source towards surface gateway.

(19) $$\;Min\sum_{d_{S-G}=d_{0}}^{d_{maximum}^{S-G}}\;Thermal\;Noise,\;\;\;\;\;\forall \;d\;\epsilon \;R,$$

(20) $$\;Min\sum_{d_{S-G}=d_{0}}^{d_{maximum}^{S-G}}\;Shipping\;Noise,\;\;\;\;\;\forall \;d\;\epsilon \;R,$$

therefore, noise should be minimum to get the maximum throughput. Meanwhile, the total noise will be the sum of both thermal and shipping noise.

#### Graphical Analysis of Throughput

Let us consider a scenario in which frequency is up to 1000 Hz with ‘$d_{S-G}$’ of 100–2000 m and noise level is up to 60 dB. Therefore, calculated thermal and shipping noise ranges from (0.037–0.75) and (0.02–0.4) dB. Therefore,

- 0.037 + 0.02 ≤ Thermal + Shipping Noise ≤ 0.75 + 0.4
- 0.037 ≤ Thermal Noise ≤ 0.75
- 0.02 ≤ Shipping Noise ≤ 0.4

**Figure 15:**
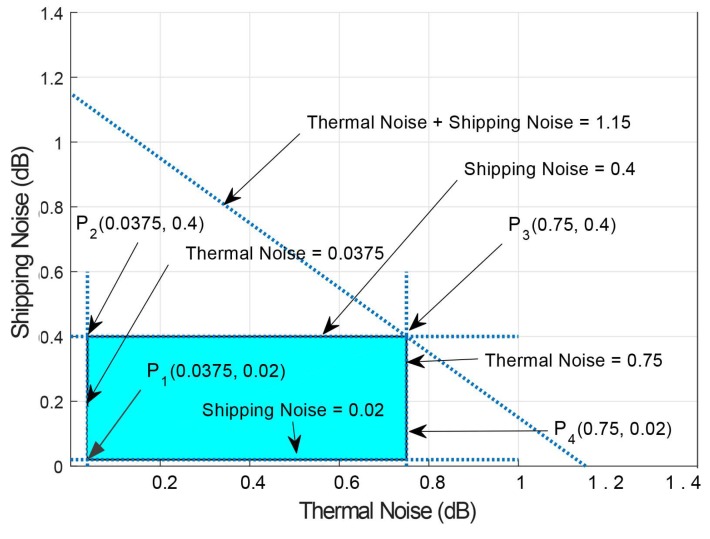
Feasible Region for Noises in UWSNs.

Feasible region of both noises are shown in Figure 15. Points $P_{1},P_{2},P_{3}$ and $P_{4}\;$ cover the corner points and provide a valid solution. Minimum the noise is the maximum be will be the packet delivery at surface gateways due to minimum error in data packets.

### 4.5. Performance Trade-Off

In this subsection, the performance of the existing and the proposed protocols are compared. To avoid the repetition, parameters are explained as general because of similar behaviors in the proposed protocols. To achieve the data reliability in data transmission, multiple copies of data packets are generated. Existing protocols experience the least delay with high EC, a high number of active nodes and high PRR. Meanwhile, proposed algorithms achieved reliable data delivery with the least EC and a minimum number of active nodes and affordable PRR. In the proposed protocols, the delay is compromised over EC. Moreover, performance trade-off and achievements with compromised parameters of existing and proposed protocols are shown in Table 10.

In the proposed protocols, a proactive approach is used to search the best shortest path, but a trade-off occurs between the EC and the delay. If the proposed protocols dissipate minimum EC to prolong the network lifespan, the network must compromise on delay, i.e., the network must pay the cost of delay. The BFS-SPF-Three and SPF-Three selects the single efficient shortest path with minimum EC which results in collision of data packets and delay. Retransmissions deliver the data packets as soon as possible which results in the least number of active nodes with minimum EC. Therefore, the lifetime of the network increases. However, the network must compromise on delay to minimize the EC.

## 5. Conclusions and Future Work

Minimum EC is one of the prime requirements in designing routing protocols in UWSNs because of limited resources. The random distribution of nodes, void holes and a decrease in packet drop ratio decreases the lifespan of the network. In this paper, two routing protocols: SPF-Three and BFS-SPF-Three are proposed to attain the reliability in data transmission and energy efficiency using ’Dijkstra’ algorithm (a greedy approach for SPF). In both proposed protocols, the binary tree generation starts from a noisy layer to minimize the effect of the existing noises in data packets to get attenuated. This binary tree generation increases data reliability with minimum EC.

The proposed protocols use proactive approach for opportunistic routing to achieve the energy efficiency, RPER and reduce the number of active nodes to prolong the network lifespan. The simulation results show the efficiency of the proposed protocols in terms of minimum EC. The number of active nodes and RPER are also minimized.

In future, we will explore and implement these routing protocols with some machine learning and artificial intelligent techniques to avoid void holes. To implement these techniques on test bed for getting more precision in results, will be the new direction of our research. Idea about implementation of “Internet of things” is also under consideration.

## Figures and Tables

**Table 1:** Abbreviations.

Descriptions	Abbreviations
Underwater Wireless Sensor Networks	UWSNs
IoT-enabled Underwater Wireless Sensor Networks	IoT-UWSNs
Energy Consumption	EC
Forward Layered Multi-path Power Control One	FLMPC-One
Forward Layered Multi-path Power Control Two	FLMPC-Two
Layered Multi-path Routing Control protocol	LMPC
Shortest Path First	SPF
Breadth First Search	BFS
Packet Received Ratio	PRR
Bit Error Rate	BER
Required Packet Error Rate	RPER
Noise Resources	NR
Noise Power	NP
Signal to Noise Ratio	SNR
Packet Error Rate	PER
Packet Delivery Rate	PDR
Distance from *n* − 1 to $n_{th}$ node	D
Transmission Power	TP
Total Number of Data Packets	N
Total Hop Counts	THCs
Sparsity-aware Energy-Efficient Clustering	SEEC
Circular Sparsity-aware Energy-Efficient Clustering	CSEEC
Circular Depth-based Sparsity-aware Energy-Efficient Clustering	CDSEEC
Reliable Energy-Efficient Pressure-Based Routing	RE-PBR
Spherical Hole Repair Technique	SHORT
Geographic Forwarding based on Geo-spatial Division	GFGD
Greedy Geographic Forwarding based on Geo-spatial Division	GGFGD
Geographic and Opportunistic Routing with Depth Adjustment	GEDAR
Geo-Opportunistic Routing	GOR
Energy-Efficient Multi-hopping Routing	EAMR

**Table 2:** Acronyms.

Definitions	Symbols
Absorption Coefficient	α(F)
Binary Entropy Function	$H_{2}$
Transmission Energy	$\left(E_{t}\right)$
Receiving Energy	$\left(E_{r}\right)$
Reception Power	$P_{r}$
Total Energy	$E_{Total}$
Vertices	*V*
Channel capacity	C
Bandwidth	B
Path	*P*
Frequency	F
Packet Length	PL
Packet Size	PS
Data Rate	DR
Initial Energy of Network	$E_{Initial}$
Packet Type	P(t)
Total Time of Transmission	$t_{tot}$
Transmission Power	$P_{t}$
Data Packet	DP
Total Leftover Energy of the Network Nodes after Forwarding the Data Packets	$E_{t}^{remaining}$
Total Receiving Energy of the Network Nodes after Receiving the Data Packets	$E_{r}^{remaining}$

**Table 3:** Summarized Literature Review.

Names of Protocol (s)	Features of Protocol (s)	Achievements of Protocol (s)	Limitation of Protocol (s)
GEDAR [9]	Multi-hoping	Void hole coverage	High EC during mobility in nodes
HYDROCAST [10]	Multi-hoping	High packet delivery ratio with Reliable transmission of packets	High delay and EC
LMPC [11]	Multi-hoping and Binary tree generation	High packet delivery ratio with minimum delay	Void holes with long term transmission
FLMPC-one and FLMPC-two [12]	Multi-hoping and Binary tree generations	High delay, high packet delivery ratio, low EC and High packet received ratio	Sparse regions effect its processing time
WDFAD-DBR [13]	Multi-hoping	Less delay and high packet delivery ratio	Void holes and wastage of energy with long communication
GFGD and GFGD(GGFGD) [14]	Multi-hoping between small cubes	Energy efficacy and least delay	No
SHORT [15]	Multi-hoping	High throughput of network and long life of network	High delay
RE-PBR [16]	Multi-hoping	Energy efficient and reliability in data transmission	Sparse regions effects the processing time of network
OR [17]	Opportunistic routing	Interference avoidance	Trade-off between energy and distance is not discussed
GDGOR [18]	Multi-hoping and depth adjustment	Void node avoidance	Trade-off between high EC is persisted
SEEC, CSEEC and CDSEEC [19]	Clustering in the square and circular fields	Energy minimization is achieved successfully	Distance dependency on energy is not considered and communication overhead is not focused
EAMR [20]	Multi-hoping	Reduction in excessive overhead and reduce cluster head changes with increase in network lifetime	In sparse region, cluster heads cannot bear the load

**Table 4:** Simulation Parameters.

Parameters	Values
Area	2000 m × 2000 m
Noise of Ship	0.2 db
Wind	5 m/s
Number of Nodes	150
Number of Cross Nodes	25
Sinks	5
Layers	6
Number of Iterations	10
Frequency	914 × 10${}^{6}$ Hz
Total Energy	1000 J
Initialized Energy per Node	0.667 J
Transmission Power	0.66 mW
Receiving Power	0.035 mW

**Table 5:** EC Values with PS of 100 bytes.

EC	PS = 100 bytes		
	DR = 10 Kbps	DR = 20 Kbps	DR = 30 Kbps
FLMPC-One	343.2756	192.7555	161.8370
FLMPC-Two	214.0730	157.0365	138.0243
SPF-Three	123.1215	111.5607	107.7072
BFS-SPF-Three	80.8360	67.4147	62.9410

**Table 6:** EC Values with PS of 200 bytes.

EC	PS = 200 bytes		
	DR = 10 Kbps	DR = 20 Kbps	DR = 30 Kbps
FLMPC-One	471.0220	287.6480	223.6740
FLMPC-Two	328.1460	214.0730	176.0487
SPF-Three	146.2430	123.1215	115.4143
BFS-SPF-Three	107.6785	80.8360	71.8885

**Table 7:** EC Values with PS of 300 bytes.

EC	PS = 300 bytes		
	DR = 10 Kbps	DR = 20 Kbps	DR = 30 Kbps
FLMPC-One	546.6355	435.8860	287.0280
FLMPC-Two	442.2190	271.1095	214.0730
SPF-Three	169.3645	134.6822	123.1215
BFS-SPF-Three	134.5210	94.2572	80.8360

**Table 8:** EC Values with PS of 400 bytes.

EC	PS = 400 bytes		
	DR = 10 Kbps	DR = 20 Kbps	DR = 30 Kbps
FLMPC-One	623.4360	477.0100	420.3573
FLMPC-Two	556.2920	328.1460	252.0973
SPF-Three	192.4860	146.2430	130.8287
BFS-SPF-Three	161.3635	107.6785	89.7835

**Table 9:** EC Values with PS of 500 bytes.

EC	PS = 500 bytes		
	DR = 10 Kbps	DR = 20 Kbps	DR = 30 Kbps
FLMPC-One	696.3850	521.2662	454.5842
FLMPC-Two	649.9825	389.2062	290.1217
SPF-Three	215.6075	157.8037	138.5358
BFS-SPF-Three	188.2060	121.0997	98.7310

**Table 10:** Performance Trade-offs between Existing and Proposed Protocols.

Protocols	Parameters	
	Achievements	Compromised Parameters
FLMPC-One	Less delay	High EC, active nodes and PRR
FLMPC-Two	Less reliability and delay	High EC, active nodes and PRR
SPF-Three	High reliability, less EC, less active nodes, and affordable PRR	Delay
BFS-SPF-Three	High reliability, less EC, high active nodes, and affordable PRR	Delay
